# The potential consequences of ‘bee washing’ on wild bee health and conservation

**DOI:** 10.1016/j.ijppaw.2022.03.011

**Published:** 2022-04-02

**Authors:** Sheila R. Colla

**Affiliations:** Faculty of Environmental and Urban Change, York University, Canada

## Abstract

Concern around declining bee populations globally has become an environmental issue of mainstream importance. Policymakers, scientists, environmental non-government organizations, media outlets and the public have displayed great interest in conservation actions to support pollinators. As with many environmental causes, green washing, or in this case ‘bee washing’, has become rampant. Bee washing can lead to multiple negative consequences, including misinformation, misallocation of resources, increasing threats and steering public understanding and environmental policy away from evidence-based decision-making. Here I will discuss the multiple potential consequences of bee washing on efforts to conserve declining wild bees and promote wild bee health.

## Highlights

• Concern around declining bee populations globally has become an environmental issue of mainstream importance.
• Policymakers, scientists, environmental non-government organizations, media outlets and the public have displayed interest in conservation action to support pollinators.
• ‘Bee washing’, has become rampant.
• Narratives and actions tend to focus on low-hanging fruit, actions which are easy to address and/or the selling of commercial items where industry benefits but the species of concern do not.
• Negative consequences include misinformation, misallocation of resources, increasing threats and steering environmental policy away from evidence-based decision-making.

## Graphical abstract

**Figure:**
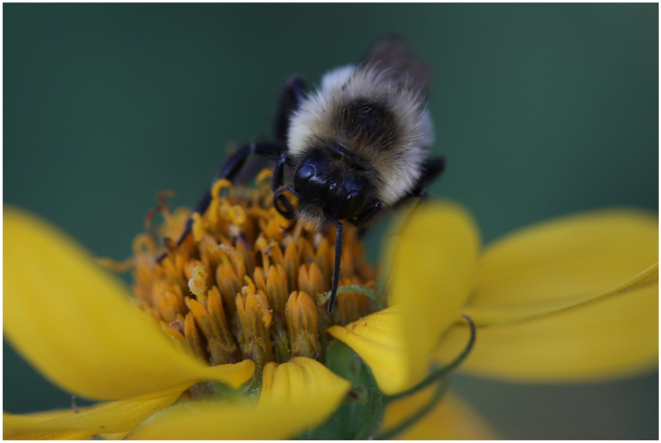

Over recent decades, the plight of wild bees and other pollinators has gone from a niche area to one of the most mainstream environmental topics (Hall and Martins, 2020). Scientific research interest, capacity and consequently publications have grown tremendously in recent years, as has mainstream media coverage (Smith and Saunders 2016; Wignall et al., 2019; Schatz et al., 2021). Political, industry and ENGO actions have also ramped up, reflecting, and capitalizing on the general public's growing interest. The public has been engaged in a variety of ways to “save the bees” including through community science (Schatz et al., 2021; MacPhail et al., 2020), policy consultation (Nicholls et al., 2020), planting of pollinator gardens (Wignall et al., 2019) and other widespread initiatives and campaigns. While generally understudied, understanding these and the other human dimensions of pollinator conservation are critical to effectively move towards the overarching goals of conserving bee biodiversity and supporting wild bee health (Hall and Martins 2020).

‘Bee washing’, a term coined by MacIvor and Packer (2015), refers to items or actions claiming to support declining bee populations, and thus claiming to be pro-environment, without due diligence or scientific support. MacIvor and Packer (2015) use the example of the widespread promotion and sale of bee hotels as an initiative to increase wild bee populations which is not backed by science (MacIvor and Packer 2015). Since their publication, the market has been flooded with additional bee washing initiatives, including the well-known multi-million dollar crowd-sourcing campaigns for the ‘Flow hive’ (Marcum and Blair 2017) and sustainability initiatives involving rooftop honeybee hives, often concentrated in cities (Casanelles-Abella and Moretti 2022) and associated with businesses (Egerer, M. and Kowarik, I., 2020). These initiatives tap into the public's concern for pollinator decline but are often based on misconceptions or economic priorities, such as focusing on “saving” the honeybee, a managed species not at-risk of extinction (Colla and MacIvor 2017; Casanelles-Abella and Moretti 2022).

The crux of the matter is that when conservation actions are misplaced, as in the case with bee washing, they can actively harm populations meant to be conserved, waste limited resources (e.g. time, energy, money), misinform the public and/or de-legitimize scientific evidence (Ford et al., 2021). Misplaced conservation can intentionally prioritize short-term gains for people and industry instead of long-term benefits to declining wildlife species and shows the need for processes that elevate the role of science and evidence-based decision-making in policy, ENGO programming and other spheres (Ford et al., 2021). The prevalence of misinformation can also cause division between stakeholders, reduced credibility of scientists and create other conflicts which make evidence-based decision-making more challenging (e.g. Loss and Marra 2018). In the case of bees, bee washing can often focus on low hanging fruit, individual versus systemic change, feel-good actions, and actions less in conflict with industry. These actions likely rely on overly simplified information or even intentional misinformation and have repercussions in the understanding of the issues by the public, media and policymakers. Misinformed actions (e.g. increasing honeybee hives in cities and protected areas outside of their native ranges in the name of sustainability) can move us away from the ultimate goals of protecting bee biodiversity, bee health and the ecosystem services they provide. In some cases, ‘bee washing’ may include actions which provide incrementally beneficial or neutral actions, such as the planting of pollinator gardens, but these can move resources, attention and political opportunities away from addressing more dire threats or increase misunderstanding of threats (Ford et al., 2021). In a Canadian study, the overwhelming majority of interviewed participants believed the loss of flowers and the use of pesticides were the main threats to declining bees in Canada (van Vierssen Trip et al., 2020). Unsurprisingly, these two issues also dominate pollinator protection policy (e.g. Bloom et al., 2021; Schatz et al., 2021; Nicholls et al., 2020) despite not having strong scientific support as the only or main drivers for declining bee populations and health (Dicks 2013; Williams and Osbourne 2009). This kind of inaccurate information combined with the conflation of livestock management issues of managed bees with threats to wild bees reframes economic costs as ecological costs (Senapathi et al., 2015).

Bee washing initiatives which promote managed species contribute to one of the main threats to wild bee health. The spillover of pathogens from managed species to closely related species has been documented in a variety of taxa, often with devastating consequences for wild populations (Daszak et al., 2000). Approximately 15 years ago, colleagues and I first documented the phenomenon of pathogen spillover in North America from managed bumblebees to wild bumblebees foraging near greenhouses where managed bees were being used (Colla et al., 2006). Similar observations have since been made elsewhere around the globe and the negative impacts on wildlife are of concern to scientists (Graystock et al., 2016; Kojima et al., 2011). However, pinpointing pathogen spillover from managed animals as a main cause of wildlife decline is incredibly challenging. Often, the evidence becomes apparent only once the damage has been done and populations have dwindled. This is particularly challenging for insect populations where standardized and widespread monitoring is rare.

There are multiple lines of evidence that pathogen spillover indeed is a main threat to multiple at-risk bumblebee species and thus the precautionary principle should be used. The precautionary principle contends that when it is scientifically plausible that human activities may lead to morally unacceptable harm, actions shall be taken to avoid or mitigate that harm: uncertainty should not be an excuse to delay action (Drivdal and van der Sluijs 2021.).Where studied, vulnerable species have shown to have higher levels of pathogens (Cordes et al., 2012; Cameron et al., 2011) and evidence of pathogen exposure (Cameron et al., 2016; Kent et al., 2018; Tsvetkov et al., 2021). In addition, evidence is growing for the spillover of diseases from managed honeybees with wild bees and other wild insects (Alger et al., 2019; Bailes et al., 2018; Fürst et al., 2014; Tapia-González et al., 2019) These diseases can have detrimental impacts on species newly exposed to the but also indicate an ongoing potential for a novel zoonotic event which could negatively impact currently common and stable species as managed bees continue to proliferate. Recently, a global-scale study found virus levels of bumblebees and solitary bees to be positively related to the viral prevalence of co-occurring honeybees (Piot et al., 2022). Additionally, other species of bees are increasingly being sold and distributed outside of their native ranges for hobby and commercial use (e.g. *Osmia, Megachile*), often under the bee washing guise of solitary bee support, and their disease dynamics are completely unstudied (Evison and Jensen, 2018). For example, LeCroy et al. (2020) discuss the potential of disease spillover to be a possible unstudied mechanism explaining the rapid increase of the non-native *Osmia cornifrons* and declines of native *Osmia* populations in North America.

While there are several other documented and suspected drivers of wild bee decline globally, the proliferation and widespread use managed bees has become an issue of increasing conservation concern and one that bee washing activities may exacerbate. With the increase in mainstream concern about bees, the popularity of managed honeybees has also increased in urban areas as part of “sustainability” initiatives (Baldock 2020). A recent study in Switzerland found city hive densities to have risen in recent years and raised concerns about the sustainability of this practice given the limited availability of forage resources (Casanelles-Abella and Moretti 2022). The use of the European honeybee (*Apis mellifera*), the Buff-tailed Bumblebee (*Bombus terrestris*) as well as other managed bee species outside their native ranges has resulted in multiple negative impacts to local bee fauna associated with competition and invasion. Hives of bumblebees and honeybees are moved around within and between countries and the escape of bees and interactions with local fauna are not monitored (Aizen et al., 2019). Numerous studies have documented negative impacts associated with bee communities being dominated by managed species (e.g. Garibaldi et al., 2021; Renner et al., 2021; Russo 2016) and devastating impacts for some wild species (e.g. Schmid-Hempel 2014; Arbetman et al., 2013; Morales et al., 2013; Dafni et al., 2010). This is an ongoing, self-reinforcing and growing threat to native bee biodiversity (Russo et al., 2021), especially as demand for pollination services increases (Aizen and Harder 2009) and quality of natural habitat continues to degrade.

In order to better protect wild bee health and biodiversity, conservation actions and policy will have to shift away from bee washing to more evidence-based, nuanced and precautionary approaches. Efforts should focus on reducing the reliance of systems on managed bees and reducing the impacts of managed bee use on wild bees. This will require policy which acknowledges and values the importance of wild bee health biodiversity for pollination services for crop plants and for resilience under climate change. Critical actions to protect wild bee health includes screening and monitoring of pathogens among commercial stock and in adjacent wild populations. Legal frameworks should be developed for the restrictions of bee movement outside of native ranges (Aizen et al., 2019; Orr et al., 2022) and density limits where non-native species are already established (Goulson and Sparrow 2009). Working agricultural lands should be designed to incorporate habitat and reduce the use of pesticides to best support wild bees (e.g. Garibaldi et al., 2021a, Garibaldi et al., 2021b; Kovács-Hostyánszki et al., 2017), perhaps reducing long-term demands for managed bees for ecosystem services.

It is increasingly understood that human dimensions of conservation issues can have a significant impact on conservation outcomes (Bennett et al., 2017). Law, policy, education, economics and other social spheres need to be integrated when planning for effective conservation, particularly when considering the socio-ecological problem of pollinator decline (Mupepele et al., 2021; Hall and Martins, 2020). Bee washing can set back momentum around pollinator conservation through the spread misinformation, by increasing key threats and by focusing on actions that financially benefit businesses or organizations. While research on most of the world's 20,000 + bee species is largely lacking, the evidence we do have points to a complex issue with a variety of context-dependent threats causing declines in wild bee health and populations (LeBuhn and Vargas 2021; Williams and Osborne 2009). Conserving native pollinator diversity will increase resiliency in light of climate change and other environmental change (Senapathi et al., 2015) and increase food security, especially in areas where renting pollination services possible due to financial constraints. Going forward, scientists, environmentalists, science writers, public servants and other groups should be critical of messaging around bee conservation and conservation actions which may be deemed examples of bee washing. Solutions should be evidence-based and take into consideration the role of socio-cultural factors which may influence conservation outcomes. As keystone species, wild bees are critical components to the ecological integrity of natural ecosystems and the wildlife they support, and all available resources should be directed to better collective understanding and evidence-based conservation action.

## Declaration of competing interest

None.
